# Using mixed methods for genetic counseling research

**DOI:** 10.1002/jgc4.70031

**Published:** 2025-04-30

**Authors:** Kennedy Borle, Jehannine (J9) Austin

**Affiliations:** ^1^ Interdisciplinary Studies Program, Faculty of Graduate and Postdoctoral Studies University of British Columbia Vancouver British Columbia Canada; ^2^ Department of Psychiatry, Faculty of Medicine University of British Columbia Vancouver British Columbia Canada; ^3^ Department of Medical Genetics, Faculty of Medicine University of British Columbia Vancouver British Columbia Canada

**Keywords:** knowledge, methodology, mixed methods, rigor

## Abstract

Mixed methods research encompasses methodological approaches that involve the collection, analysis, and integration of qualitative and quantitative data. Mixed methods are useful for complex research questions, applied research settings, and when end users value multiple forms of evidence, which makes mixed methods suitable for many areas of genetic counseling research. High‐quality rigorous research methods are required to generate useful knowledge that can advance the field of genetic counseling. The goal of this paper was to provide an introduction to mixed methods research and discuss the rationale, research paradigms, study designs, methodological considerations, opportunities, and challenges in genetic counseling research.

**What is known about this topic:**

Mixed methods research combines qualitative and quantitative approaches to provide a comprehensive understanding of different research phenomena. There is guidance about how to conduct rigorous mixed methods research to increase the quality and validity of research findings.

**What this paper adds to the topic:**

This paper seeks to provide an introduction to mixed methods approaches by collating and applying common mixed methods practices to the field of genetic counseling.

## 1 INTRODUCTION

“Mixed methods” is an umbrella term used to describe a set of research methods that involve the collection, analyses, and integration of both quantitative and qualitative data within the same study (Shorten & Smith, 2017). Genetic counseling research encompasses a broad range of topics including but not limited to research about patient care, health systems and services, genetic counseling workforce and professional issues, training and education, measurement and implementation science, and the process and outcomes of genetic counseling (Austin, 2024). Across all these broad topic areas, there are many situations where mixed methods approaches could be the most appropriate methodology to generate evidence. The goal of this paper was to describe some of the key considerations around conducting the kind of rigorous mixed methods research that can generate valid and useful evidence in the context of genetic counseling research.

## 2 DEFINITION OF MIXED METHODS RESEARCH

Mixed methods research has emerged as a unique approach with its own methodological considerations and techniques that incorporate both qualitative and quantitative elements, but is not simply the combination of these two methodological approaches.

Creswell and Plano Clark define the core characteristics of mixed methods research as (Creswell & Plano Clark, 2018):

- collecting and analyzing qualitative and quantitative data,
- integrating the data and results,
- conducting research according to rigorous study designs that describe the procedures and rationale for the study, and
- grounding the research in applicable theories and philosophies.

Mixed methods research allows researchers to use different types of tools to answer questions that are not adequately answered through the use of either qualitative or quantitative approaches alone (Creswell & Tashakkori, 2007). It encourages researchers to use multiple paradigms and can be the bridge between the constructed dichotomy of qualitative and quantitative approaches (Creswell & Plano Clark, 2018).

Common applications of mixed methods research include:

- explaining or exploring initial results from the use of a single methodological study,
- psychometric instrument creation or validation,
- multi‐part studies that involve the development, implementation, and evaluation of a program or intervention, and
- studies that have multiple end users who value different types of evidence (Creswell & Plano Clark, 2018).

The use of mixed methods can either be planned from the outset of a study (fixed study design) or can develop over the research process to further explore incomplete results or investigate new insights (emergent study design) (Creswell & Plano Clark, 2018). The qualitative and quantitative approaches are often referred to as “strands” and they can have differing relative priorities depending on the study goals (Creswell & Plano Clark, 2018). Many of the examples of mixed methods genetic counseling research studies prioritize the quantitative strand; however, there are examples of qualitatively oriented mixed methods research and studies where the strands are given equal priority to answer a given research question (Creswell, 2014; Poth & Shannon‐Baker, 2022).

## 3 MIXED METHODS AND GENETIC COUNSELING RESEARCH

Mixed methods research has been growing in popularity in healthcare research (Askun & Cizel, 2020; Plano Clark, 2010; Wasti et al., 2022). An exploration of the use of mixed methods research in NIH applications found that the number of proposals using this approach increased nearly exponentially from 1997 to 2008, from one proposal in 1997, to 60 in 2008 (Plano Clark, 2010). This increase is likely in response to the increased complexity of healthcare related research questions and the understanding and growing acceptance that multiple forms of evidence are required to generate useful knowledge for diverse groups of end users (Poth & Shannon‐Baker, 2022). Despite this growing use of mixed methods research in healthcare, there are only seven articles within the *Journal of Genetic Counseling* that include the term “mixed methods” among their keywords. While this is a crude search that does not capture all genetic counseling literature, it does suggest that mixed methods approaches are not yet widely used—or acknowledged as being used—in research in this field (Wasti et al., 2022). Given that mixed methods approaches would theoretically be ideally suited to generating useful knowledge in the context of the inherent interdisciplinarity and complexity of the field of genetic counseling, there are several plausible explanations for its low rate of appearance in this journal. The first is that genetic counseling research is a relatively new field of study, and because mixed methods research requires a researcher to have expertise in both qualitative and quantitative methodologies, there may be limited expertise in the field to conduct mixed methods research. Second, a large proportion of genetic counseling research is conducted as directed studies or capstone thesis projects by students who are completing their Master's degree in Genetic Counseling (Zayhowski et al., 2024). Such projects are typically smaller in scope than a full thesis research project and the programmatic time constraints are not typically conducive to mixed methods research designs that involve multiple data collection and analysis steps. Third, it is possible that mixed methods research is being conducted at a higher rate than ultimately makes it to be published in academic journals, perhaps because they are not being submitted, or perhaps despite being submitted, mixed methods may be viewed less favorably by journals and/or reviewers, or these studies are not meeting quality thresholds.

However, as the field of genetic counseling research evolves and strives for increased academic credibility (Austin, 2024), there is a need for researchers to employ all research methods available to produce useful knowledge that can benefit clinical practice and the field as a whole. The logistical barriers around time and student research are likely to persist in the short term, but the lack of expertise can be addressed through the creation of tailored education or practical guidance documents for genetic counseling researchers.

## 4 RATIONALE FOR USING MIXED METHODS

Mixed methods research is often suitable for complex research questions or contexts where integrating multiple forms of evidence would lead to increased value above using a single type of methodological approach. There are many different rationales to justify the use of mixed methods research approaches, the basis for all of them is to be able to expand on the breadth and/or depth of understanding of the phenomenon being studied. Table 1 provides a summary of the goals of using mixed methods approaches. For example, Hesse‐Biber et al., provided this rationale for using mixed methods “employing a mixed methods design allowed for the use of qualitative data in a targeted manner in order to gain a better understanding of how the gender differences uncovered by the survey results were experienced differently in the lived context of our participants and to prevent essentialism of the survey results” in their study seeking to explore how gender contributed to differences in decision making for genetic testing and post‐test health behaviors (Hesse‐Biber, 2018).

**TABLE 1:** Reasons to use mixed methods.

Goal	Description	Notes
Triangulation or convergence	Seeks to corroborate knowledge to increase the certainty of the results by incorporating evidence from multiple sources (Best et al., 2020; Doyle et al., 2016; Wong et al., 2015)	Aligns with post‐positivist/positivist orientations where the goal is to use multiple evidence sources to add credibility to findings about a single objective truth
Crystallization	Using multiple forms of evidence to more richly understand the phenomenon (Ellingson, 2012)	Aligns with interpretivist/constructivist orientations by using multiple forms of evidence as a way to explore multiple subjective realities
Complementarity	Investigating different aspects of the same phenomenon under a unifying research question (Best et al., 2020; Biasiotto et al., 2023; Peter et al., 2022)	Typically involves distinct research questions for the qualitative and quantitative strands that are unified under a broad mixed methods research question (Onwuegbuzie & Leech, 2006)
Development	Results from one methodological approach inform the next phase (Creswell & Plano Clark, 2018; Doyle et al., 2016)	Common in emergent study designs
Illustration	Qualitative data are used to provide human‐context around quantitative findings (Doyle et al., 2016)	Study designs using this rationale need to be mindful to still use rigorous procedures and integration for both of the strands
Offset weakness	Using multiple forms of evidence to minimize the weaknesses or sources of bias resulting from using a single methodological approach (Bryman, 2007; Doyle et al., 2016; Greene et al., 1989)	Refers to general sources of bias or weakness present in single methodological approaches, the researchers still need to use rigorous procedures and integration for both of the strands

## 5 RESEARCH PARADIGMS

Paradigms are the fundamental assumptions about the way the world works that guide knowledge production. They describe ontology (nature of reality), epistemology (nature of knowledge), axiology (roles and values of the research process), methodology (processes of conducting research), and rhetoric (language used in research) (Creswell & Plano Clark, 2018; Tracy, 2020). Purposefully and thoughtfully founding research within an established paradigm can allow for alignment between the research goals and processes and increase rigor, which—in turn—can increase the usefulness of the knowledge generated.

- Positivism is based on the traditional scientific method, whereby we formulate hypotheses and then deductively generate quantitative evidence that supports or refutes the hypothesis. The main goal of positivist research was to explain associations or causal relationships between variables to ultimately be able to predict the outcome of interest (Maksimović & Evtimov, 2023; Park et al., 2020). The positivist paradigm is founded on the ideas that there is a single knowable reality that can be understood and measured, knowledge can be generated objectively without influence by researchers, and that the values of the researchers and society more broadly should not interfere or interact with the production of knowledge (Maksimović & Evtimov, 2023; Park et al., 2020).
- Post‐positivism shares with positivism the same fundamental assumptions about a single knowable reality, but accepts that researchers, as human beings, are not able to objectively view the world (Maksimović & Evtimov, 2023). Post‐positivism acknowledges the existence of human subjectivity but ultimately advocates that the generation of knowledge should be as objective as possible (Maksimović & Evtimov, 2023).
- Constructivist or interpretive paradigms view reality as being socially constructed, and multiple subjective realities can co‐exist. In these paradigms, the researcher is a self‐reflective conduit for the research; they are unable to be separated from the research process (Tracy, 2020; Wainstein et al., 2023).
- Critical or transformative paradigms view the nature of reality as being constructed over time, shaped by systems of power and identity/privilege in a manner that is highly informed by history and society, subjective—and that multiple realities can therefore exist at the same time (Tracy, 2020; Wainstein et al., 2023). The goal of research in these paradigms was to improve society and bring about social and political change. The role of researchers was to consider how power and privilege impact the production and ownership of knowledge (Creswell & Plano Clark, 2018; Tracy, 2020; Wainstein et al., 2023).

The paradigms that are most often used for quantitative research are positivism and post‐positivism, and while these are also used in some qualitative research, most paradigms that guide qualitative approaches have more space for subjectivity. While mixed methods researchers can use *pluralism*, where they switch between or combine paradigms to guide the qualitative and quantitative components (or strands) of their research, one of the challenges is that there can be tensions between paradigms (Creswell & Tashakkori, 2007). In some situations, researchers may be able to find commonalities between the paradigms, such as common understandings of the need for rigor and trustworthiness (elements of epistemology) and the overarching common goals of conducting research to better understand aspects of human life, which may mitigate these tensions (Johnson & Onwuegbuzie, 2004). Alternatively, a *pragmatic* paradigm can be used for mixed methods research (Creswell & Plano Clark, 2018; Johnson & Onwuegbuzie, 2004). Pragmatism is a more practical paradigm that values using diverse approaches, objective and subjective knowledge, and prioritizing reason and function above paradigmatic purity (Creswell & Plano Clark, 2018; Feilzer, 2010). Pragmatism in mixed methods research is useful for resolving the tension between using qualitative and quantitative methods in a single study and can overcome the constructed dichotomies present between positivist and other paradigms and the reliance on either subjective or objective measurement (Creswell & Plano Clark, 2018; Feilzer, 2010; Johnson & Onwuegbuzie, 2004). Pragmatism is a highly effective paradigm for genetic counseling research because the emphasis on practicality increases the likelihood that the knowledge produced will be useful and relevant to clinical practice or health service provision (Chen & Goodson, 2009). In a mixed methods genetic counseling research study exploring individuals' experiences receiving genomic sequencing through the 100,000 Genomes Project, the authors described their paradigmatic orientation as “working within a pragmatist paradigm (a worldview that focuses on ‘what works’ rather than what might be considered absolutely and objectively ‘true’ or ‘real’…)” (Peter et al., 2022). The challenge with using a pragmatic paradigm is that because it is so highly flexible, there is less substantive guidance on the best practices for actually doing pragmatic research (Johnson & Onwuegbuzie, 2004). However, there are proposed principles for pragmatic rigor (Robey et al., 2019):

- Relevance: describes how well the problem being researched aligns with the end user priorities.
- Actionability is the degree to which the knowledge produced can be implemented into practice.
- Comprehensibility: is about the effectiveness of knowledge translation from the researchers to the end users.
- Ethical reasoning: describes the consideration of all end users and invested parties or persons and the justification of the benefits and consequences to these people and groups (Robey et al., 2019).

Because rigor is an important aspect of generating useful knowledge, these guiding principles should be observed and transparently reported on when researchers choose to use a pragmatic research paradigm.

## 6 REFLEXIVITY AND POSITIONALITY

Reflexive research practices are a critical component of conducting mixed methods research. Reflexivity is defined as an analysis of how the researchers positionality, assumptions, beliefs, and judgment systems interact with the research process (Jamieson et al., 2023). Positionality is the exploration of how identities, experiences, and values influence the way that the researcher interacts with the research (Topp et al., 2021). Reflexivity is especially important in mixed methods research, in part because there is often pluralism, pragmatism, or switching between research paradigms which inform key methodological decisions. Also, because there is a high degree of flexibility in mixed methods research, the nonlinearity of the research process is seen as inherently reflexive because the researchers are actively making decisions throughout the research process, rather than following a set of rigidly defined procedures (Jamieson et al., 2023). Practicing reflexivity is an expected part of qualitative research, and there is a growing expectation for this to be more fully integrated into quantitative research as well (Castillo & Babb, 2024; Jamieson et al., 2023). It can take the form of memoing, reflexive journaling, team discussions, and self‐reflective prompts throughout the research process (Olaghere, 2022; Topp et al., 2021; Walker et al., 2013). An example of this from genetic counseling research is a researcher identifying that she was being critical of the practices of other genetics healthcare professionals because of her own ideals about the provision of genetic testing (Hiller & Vears, 2016). Incorporating and reporting on reflexivity in mixed methods research has been identified as a gap in other fields of research; for example, in educational research, less than 10% of mixed methods manuscripts reported on reflexivity (Cain et al., 2019). Having high standards for reflexivity in genetic counseling research regardless of the methodology is important for advancing high quality, rigorous, and ethical genetic counseling research (Wainstein et al., 2023; Zayhowski et al., 2024).

## 7 STUDY DESIGNS

There has been significant discussion about the typology of mixed methods research methods with regard to how best to categorize and describe the methods being used. Researchers first need to determine if they are planning to use mixed methods from the outset (fixed study design) or if the decision to use mixed methods arose through the initial research study yielding incomplete results or leading to new insights (emergent study design). Then, to more specifically describe the mixed methodologies, there are over a dozen established typologies that can be used. Creswell & Plano Clark are some of the global experts in mixed methods research, and the typology they summarize in their textbook is commonly used in health research. Because it is straightforward and descriptive, this typology is potentially useful for genetic counseling research (Creswell, 2014; Creswell & Plano Clark, 2018). However, there are other typologies that have been proposed by Teddlie, Tashakkori, Onwuegbuzie, Johnson, and others that are valid for use in genetic counseling research as well. (Creswell et al., 2006; Johnson & Onwuegbuzie, 2004; Leech & Onwuegbuzie, 2009; Teddlie & Tashakkori, 2012) Researchers should determine which typology is most suitable for their research and follow it accordingly to ensure alignment across all stages of the research process.

Typologies to describe mixed methods research vary according to purpose, but they all seek to describe and justify, at a minimum, the:

- purpose for mixing methods,
- timing of qualitative and quantitative data collection and analysis,
- relative importance or priority of the types of data, and
- level of interaction between the qualitative and quantitative data.

Creswell and Plano Clark (2018) outline and describe the purpose, timing, priority, and level of interaction of the most common uses of three core mixed methods designs. The three core study designs for mixed methods research are convergent, explanatory sequential, and exploratory sequential. In convergent designs, the qualitative and quantitative strands of data are collected simultaneously or within the same time period, given equivalent priority, and then integrated during data analysis. In explanatory sequential designs, the quantitative data are collected and analyzed first, followed by the qualitative data collection and analysis with the goal of expanding on or explaining the findings from the qualitative data. For exploratory sequential designs, the qualitative data collection and analysis occur first, typically to inform the quantitative data collection and analytic approach. When designing a mixed methods study, it is also important to outline where and when integration is going to take place. It can occur through data collection, analytical methods (described below), and interpretation and reporting; however, mixed methods approaches that only engage in integration at the data collection stage (i.e., using one dataset to recruit participants for a subsequent research study) rarely reflect high‐quality mixed methodology (Creswell & Plano Clark, 2018; Fetters et al., 2013; Zhang & Creswell, 2013). More details about these study design types are provided in Table 2. It is also worth noting that there are deviations from these core designs, described as “complex” study designs by Creswell and Plano Clark. Complex study designs may involve multistep data collection, a combination of fixed and emergent study designs, or differences in whether the quantitative or qualitative strand is given higher priority within a core study design. For example, a researcher could plan to conduct a fixed exploratory sequential study design, where interviews are conducted to help inform items to include on a survey of patient experiences with a new genetic counseling service. Upon completion of the survey, however, the researcher identifies responses that warrant further qualitative investigation to better understand the survey. This example combines fixed and emergent designs and exploratory and explanatory sequential designs in the service of generating evidence for a cohesive research question.

**TABLE 2:** Aspects of mixed methods study designs (Creswell & Plano Clark, 2018).

Design	Purpose	Timing	Priority	Level of interaction	Examples in genetic counseling research
Convergent	Integrate complementary data on the same topic to increase understanding	Qualitative and quantitative data are collected simultaneously or in close succession and separately Data analysis is typically initially analyzed separately and then merged	The qualitative and quantitative data are given approximately equal priority or importance	Data are integrated at the analysis level with the purpose of being able to compare the findings from each arm to provide a more comprehensive level of understanding This may involve transformation of the data or other strategies to be able to compare across data types (Table 3)	Concurrent survey and interviews with individuals or families who had received exome sequencing in the prenatal or pediatric context about their actual or hypothetical preferences for receiving secondary findings from sequencing. The researchers used this approach to use qualitative data to provide depth of understanding and quantitative data to provide breadth to build confidence that the findings accurately represented the viewpoints of populations who have been historically underrepresented (Rego et al., 2022) Simultaneous data collection involving a survey and one‐on‐one interviews with *BRCA*‐positive patients to understand decision‐making processes about undergoing risk‐reducing salpingo‐oophorectomy. The goal of using this approach was to triangulate the findings from multiple data sources to yield more complete evidence toward the research question. Through synthesis of data, the authors determined that the most influential factors impacting these decision‐making processes were menopausal status and age (Casalino et al., 2023)
Explanatory sequential	Use qualitative data to explain quant results	Data collection occurs sequentially Quantitative data is collected and analyzed first, followed by qualitative data	The quantitative data are given priority, the initial orientation of the study is quantitative and the qualitative is used as a means to better explain the quant findings There are examples of sequential study designs where the qualitative strand is prioritized (Poth & Shannon‐Baker, 2022) or the two strands have more equal priority as a means of answering the specific research question	Data integration can occur at two instances The initial quant data analysis can lead to the development or refinement of the qualitative data processes (i.e., developing interview guide). There may also be integration through the sampling approach where participants in the quantitative strand are recruited for the qualitative strand Integration also occurs after the qualitative data collection to merge the two sets of connected results	An online survey followed by one‐on‐one interviews with patients who were *BRCA*‐positive to understand the impact of gender on health‐seeking behaviors. The quantitative surveys were initially used to understand trends in decision‐making and then the goal of the qualitative interviews was to explain significant differences found in the survey results, investigate outliers' experiences, and add depth to the survey findings. In this study, the quantitative results were prioritized, with the qualitative results being used to provide a richer, and more nuanced understanding of the survey findings (Hesse‐Biber, 2018) A survey and then follow‐up interviews with patients who had received genomic sequencing to understand perceptions and behaviors around family communication of genomic results. In this study, the aim of the interviews was to generate data that would expand upon survey responses. The authors found that both strands of data were highly convergent (Harrison et al., 2023)
Exploratory sequential	Use qualitative data to develop quant data collection tool or processes or select appropriate variables for further exploration	Data collection occurs sequentially Qualitative data are collected and analyzed first, then used to inform development of a quant instrument or process which is then deployed or utilized	Initially, the qualitative data are given priority, but the ultimate goal of this method is the development of a quant tool, instrument, or process which ultimately is the priority There are examples of sequential study designs where the qualitative strand is prioritized (Poth & Shannon‐Baker, 2022) or the two strands have more equal priority as a means of answering the specific research question	Integration occurs after the qualitative data analysis, as this leads directly to the development/refinement of the quant tool or instrument or process	Semi‐structured interviews were conducted with primary care providers involved in a population‐based genomic screening pilot program (DNA‐10 K) about their experiences with the pilot program. The interviews then informed a survey about the benefits, challenges, and educational needs encountered in the pilot study. The authors found that the interview findings increased the validity of the types of questions included in the survey and that the survey results lead to the ability to somewhat quantify themes in the inteviews (Lemke et al., 2020) Clinicians and researchers developed an educational pamphlet about reproductive options for people with sickle cell disease/trait. They conducted interviews with patients and used the qualitative data to revise the pamphlet and then sent the booklet to patients and conducted survey evaluation of the health literacy level of the pamphlet. Through interviews health literacy scales, and revisions, the researchers were able to greatly improve the health literacy level of the pamphlet (Early et al., 2022)

Researchers can use the elements of these study designs in flexible combinations while maintaining high rigorous standards of research as long as there is methodological coherence and clear justification for the decisions made across the research process.

Within the qualitative and quantitative strands, researchers also need strong methodological procedures for recruitment, measurement, data collection, analysis, and interpretation. The data within these strands can be generated in many different ways (not simply surveys and interviews), and researchers can consider reviewing medical records or health services utilization data, analysis of policy documents, arts‐based methods, and others as guided by the goal of the research (Creswell & Plano Clark, 2018).

Another issue that researchers should consider when planning a mixed method study design is whether a research study is truly *mixed method* or if it is better conceptualized as *multi‐method*. Sometimes the data generated from qualitative and quantitative components is far apart and difficult to transform or analyze in a format where it can be effectively integrated and used for comparison or explanation, and under these circumstances, it might be useful to consider whether it is, in fact, better suited to a multi‐method approach (Bazeley, 2016). Although mixed methods research can be suitable for many genetic counseling research topics, there is no natural hierarchy of methodologies that makes mixed methods superior to single or sequential multi‐method approaches. The “best” methodological choice is the one that will lead to the most valid, rigorous, useful evidence in a given context or for a specific research question. In fact, it can actually be harmful to prioritize using a specific methodological approach rather than to prioritize the methodology that will yield the most valid results, and researchers should be mindful of making emotional decisions to justify using mixed methods approaches.

## 8 INTEGRATION FOR ANALYSIS AND INTERPRETATION

Mixed methods data analysis consists of using the appropriate procedures to analyze the qualitative and quantitative strands and then using integration to conduct additional analyses and/or interpretations to assess how the data addresses the mixed methods research questions (Creswell & Plano Clark, 2018; Fetters et al., 2013). Hesse‐Biber et al., describe that they used a “side‐by‐side comparison for merged data analysis” for integration and they present their data in the form of “narrative weaving” to allow for comparisons between their qualitative and quantitative data (Hesse‐Biber, 2018). Harrison et al., in their study about family communication and results disclosure of genetic testing results describe their process for integration as “qualitative and quantitative data were then integrated by comparing and contrasting questionnaire responses with related qualitative responses…” In addition to integrating the data through analysis, integration can also occur through sampling, building data collection tools, and through interpretation and presentation of the results (Chen & Goodson, 2009; Fetters et al., 2013). For interpretation and presentation, analyzed data can be brought together through narrative (Biasiotto et al., 2023; Lewis et al., 2016) (descriptive writing) or through joint displays (figures, tables, or other visuals) that demonstrate the additional insights gained through using a mixed methods approach (Fetters et al., 2013). The extent and timing of data integration depends on the goals and types of data included in the study, descriptions of how to integrate data are outlined in Table 3 (Creswell & Plano Clark, 2018; Fetters et al., 2013; Onwuegbuzie & Teddlie, 2003; Zhang & Creswell, 2013).

**TABLE 3:** Ways to integrate data in mixed methods studies.

Type	Description
Data transformation	One strand of data is converted so that both can be analyzed together This may involve counting the frequencies of themes/codes present in qualitative data^a^ or transforming quantitative findings into themes or descriptions
Typology development	Analysis of one strand generates categories or a framework to analyze the other strand The results from statistical analyses could lead to the generation of deductive codes for analyzing interview transcripts
Extreme case analysis	One strand of data is used to further interrogate extreme cases found through the other strand Outliers detected during statistical analysis could be approached to conduct a follow‐up interview to further explore why they were outliers
Data correlation	Qualitative data are transformed into quantitative units and then a correlation is performed between the two strands Correlation could be used to corroborate data between the strands or demonstrate differences based on data collection mode
Data consolidation	Jointly reviewing the qualitative and quantitative data with the goal of consolidation or reduction of concepts Themes from qualitative analysis are used to focus variable selection for future quantitative studies
Data comparison	Data from different sources are compared The results from statistical analyses are compared with themes from qualitative analysis to compare and contrast the findings

^a^ Assessing the frequencies of codes is not congruent with all types of qualitative data analysis. If this is the method used for integration, ensure that it is aligned with the qualitative procedures. For example, reporting frequencies is common in content analysis but would be paradigmatically misaligned with reflexive thematic analysis (Braun & Clarke, 2024; Vears & Gillam, 2022).

Integration of the data from multiple sources will lead to the reflection about the coherence of the findings as the integration can lead to confirmation of the findings, discordance, or expansion (Fetters et al., 2013). Confirmation would indicate that the strands yielded similar results, and this could be seen as increasing the credibility of the findings (Fetters et al., 2013). This could be useful in research studies where the end users value multiple forms of evidence for decision‐making. Discordance occurs if there are conflicting findings between the strands. Researchers may evaluate methodological procedures to assess assumptions and biases or consider phenomenological or theory‐based differences for the conflicting findings. Discordant results may require future research studies (Doyle et al., 2016; Fetters et al., 2013). Expansion occurs when the findings describe different aspects of the same phenomenon in a way where the results are complementary but not completely confirmatory and having multiple sources of data leads to a richer understanding (Fetters et al., 2013). One example of a structured integration technique that uses a joint display format is called the “Pillar Integration Process” (PIP) (Johnson et al., 2019). This technique uses four stages (1) listing, (2) matching, (3) checking, and (4) pillar building. These stages are completed sequentially after data collection. In short, the PIP involves starting with either data strand and initially creating lists of the data and main categories from that strand. Then the matching stage involves analyzing the other strand and matching categories and data with the initial strand (Johnson et al., 2019). Following the matching stage, the researchers engage in checking for quality purposes to ensure completeness, accuracy, and appropriateness of the listing and matching stages (Johnson et al., 2019). Pillar building occurs by comparing and contrasting the other stages to build interpretations and explanations. The PIP technique uses a specific table design to organize and present the findings, yielding a joint display of the data as part of the analytical output. Johnson et al., provide two worked examples of this technique in health sciences (Johnson et al., 2019). The PIP is one technique that can be used to increase rigor in mixed methods research by making data analysis processes more systematic and replicable (Johnson et al., 2019).

### 8.1 Reporting mixed methods studies

There are dozens of reporting guidelines and critical appraisal tools aimed at increasing the transparency and reporting quality of mixed methods research (Fetters & Molina‐Azorin, 2019; Guetterman et al., 2023). Some of these reporting guidelines include tools that can be applied to the quantitative (e.g., STROBE, 22 items) and qualitative (e.g., COREQ, 32 items) arms, together with additional requirements for the mixing of these methods (e.g., JARS‐Mixed) (American Psychological Association, n.d.; Guetterman et al., 2023). Although reporting guidelines are a useful tool for increasing quality in research, as we have described, mixed methods research is not simply the sum of qualitative and quantitative methodologies, and reporting standards should be considered with this in mind (Guetterman et al., 2023). Critical appraisal tools for assessing the quality of mixed methods research include elements devised for assessing the quality of aspects of the study, including the rationale for using mixed methods research, a description of when and how integration occurred, the mixed method study design elements, and whether the integration was adequate or not (Heyvaert et al., 2013). In addition to describing the data collection and analysis procedures for each strand, other important minimum reporting standards include the order of data collection, which strand was considered dominant, whether the study design was fixed or emergent, and the research paradigm(s) used to situate the research (American Psychological Association, n.d.).

## 9 CHALLENGES AND CRITIQUES OF MIXED METHODS RESEARCH

As described, there are significant benefits to using mixed methods research in the context of genetic counseling research. The use of multiple forms of data better reflects the way we experience the world and may yield more comprehensive and useful knowledge about complex problems. However, there are challenges to conducting mixed methods research that are important to discuss, and we outline some of these key challenges for genetic counseling research in Table 4. The main logistical challenges are that mixed methods require more time and resources than single method studies because of the multiple forms of data collection and analysis (Creswell & Plano Clark, 2018; Dawadi et al., 2021). For example, for research questions that require rapid evidence generation to inform immediate policy decisions, the time required for sequential data collection may make such study designs unsuitable. This type of design may also be unsuitable for research that has external timeline constraints, like student research projects or—in some cases—grant funding cycles. For mixed methods studies that involve concurrent data collection, the total time required may be similar to single method studies; however, the intensity of effort during data collection and analysis would be higher. In terms of resource investment to output ratio, mixed methods research may not always be supported by granting agencies, and in a culture that values quantity of publications, a single publication as an output of the combination of two different sets of methods may not always be prized (O'Cathain et al., 2009).

**TABLE 4:** Issues when considering the use of “mixed methods” in genetic counseling research.

Category	Issue	Description	Consequence	Options
Methodological incoherence	Using mixed methods when multi‐method would be more appropriate	Using a mixed methods approach when a single or multi‐method approach would be more suitable for a given research question	Lack of methodical alignment can reduce the overall quality of evidence generated that can lead to downstream consequences as this evidence is translated into changes in genetic counseling practice	Practice reflexivity through the research process to critically examine the values behind decision‐making Have check in points to ensure alignment between the goals, questions, methods, and impacts of the research Adapt the methodological approach as needed—for example, if use of mixed methods was initially planned, but as the project unfolds, it becomes clear that is not suitable
	Incomplete integration of strands	Limited or incomplete integration of the qualitative and quantitative strands	Incomplete integration can reduce the overall quality of the evidence generated by not executing this methodology to its full potential Can also be a consequence of trying to use mixed methods when a multi‐method approach would be more appropriate	Determine if using mixed methods is aligned with the study purpose Consider employing additional strategies and time points to more fully integrate the strands Transparently report the goals, strategies, and outcomes of integrating the strands
Limited rigor in one strand	Demographic or recruitment survey for a primarily qualitative study	Administering a pre‐survey for the target population to collect demographic information and inform the sampling procedures for a primarily qualitative research study	Depending on the study, this may result in the quantitative strand having insufficient rigor, which may then end up negatively impacting the validity of the qualitative results Improperly categorizing research types can have a detrimental impact on the credibility of genetic counseling research as a whole	Create detailed analysis plans for the quantitative and qualitative strands and use best practices as guidance by referring to reporting standards, critical appraisal tools, and other published reputable sources. Consider the sample size of the survey data and whether it would be adequately powered for statistical analysis, if applicable (Chow et al., 2008; Pourhoseingholi et al., 2013). The analysis plan should also include a description of how, when, and why the data are being integrated Engage in reflexive research practices to determine if a mixed methods study design is suitable for the research purpose or if a multi‐method or single methodology design would be more appropriate Administering recruitment/demographic surveys in the context of a primarily qualitative study is a valid approach to assist in data collection and inform sampling procedures (e.g., purposive). If this is the case, conduct the study as a qualitative study and report the use of the survey for the purposes it was used for within the context of a qualitative study
	Open‐ended questions on a primarily quantitative survey (or other form of quantitative data collection)	Administering a primarily quantitative survey (or other form of quantitative data collection) that includes open‐ended questions for participants to provide longer form responses with the plan of conducting qualitative data analysis	Depending on the study, there may be insufficient data to conduct formal qualitative analysis or the researchers may not have the skills or time to conduct formal qualitative data analysis which could lead this strand to have insufficient rigor If the qualitative strand is not strongly grounded in methodology, then this would not be a mixed methods study and improperly categorizing research types can have a detrimental impact on the credibility of genetic counseling research as a whole	Create a detailed a priori plan for analyzing the qualitative data, consider the concept of information power and how it will be determined that you have enough data and that the data are rich enough for sample sufficiency (Malterud et al., 2016). Consider what steps you could take if you determine you do not have an adequate sample size to conduct qualitative analysis. This analysis plan should also describe how and why the data are being integrated If the barrier to conducting the analysis is due to limited expertise of the study team, consider diversifying the study team or consulting an expert in that area Engage in reflexive research practices to determine if a mixed methods study design is suitable for the research purpose or if a multi‐method or single methodology design would be more appropriate
Student research projects	Limited resources in the context of student research projects	Insufficient time to collect and analyze data from multiple sources within the time restrictions and competing priorities within GC training programs	Rushed execution of the research can lead to reduced quality of the research, increase likelihood of mistakes, biases, and lower validity of the findings Can also create unrealistic expectations and undue pressure for the graduate student and can negatively impact training experiences and reduce the likelihood of future participation in research	Map out the scope and necessary resources for mixed methods research from the outset Create a plan for the research if it extends beyond the program length that includes an agreement on the minimum requirements to graduate if the project is incomplete Consider employing a legacy supervision model where one student leads one of the strands and then supervises a subsequent student to lead the second strand or a multi‐year student research project where the students are collaborators (Lowes et al., 2023; Peck et al., 2022)

There may also be challenges related to rigor and trustworthiness: The use of flexible methodologies and pragmatic paradigms is often necessary to accomplish mixed methods research, but there needs to be sufficient adherence to methodological best practices to ensure that the research findings are valid (Creswell, 2014; Creswell & Plano Clark, 2018). This can be addressed through fostering genuine interdisciplinary collaborations that include individuals with expertise across research methodologies, practicing reflexivity through documenting decision‐making processes, and clear, transparent reporting of the methods involved, and to integrate reflexive research practices into the research process (Bowers et al., 2013). Genetic counseling researchers using mixed methods should be aware of these challenges and critiques and strive to conduct research that is high quality, rigorous, and valid to increase the usefulness of the knowledge produced through these approaches.

## 10 CONCLUSION

Evidence generated through research is essential for genetic counseling practice, training and education, and advancing the genetic counseling profession. In order to further the credibility of genetic counseling research, it is important that researchers are able to undertake high‐quality, methodologically rigorous studies across all types of study designs. Mixed methods approaches have the potential to generate useful knowledge for complex research questions and applied settings (like genetic counseling research) because they allow the researchers to explore a phenomenon in a way that mirrors reality, rather than splitting it into component qualitative and quantitative parts. This paper aims to serve as a foundational guide to mixed methods genetic counseling research with the goal of elevating the methodological rigor and increasing the validity of the evidence that is critical to the field of genetic counseling.

## AUTHOR CONTRIBUTIONS

KB contributed to conceptualization, analysis, writing – original draft, writing – review & editing, visualization, and project administration. JA contributed to conceptualization, analysis, writing – original draft, writing – review & editing, visualization, and supervision. The authors take responsibility for the integrity of the data and the accuracy of the manuscript and agree to be accountable for all aspects of the work in ensuring that questions related to the accuracy or integrity of any part of the work are appropriately investigated and resolved.

## CONFLICT OF INTEREST STATEMENT

The authors have no financial conflicts of interest to disclose.

## ETHICS STATEMENT

Human studies and informed consent: This manuscript did not include data from participants, and informed consent was not required.

Animal studies: This manuscript did not include data from animal studies.

## Data Availability

There was no original data generated through this manuscript.
